# Visceral pain: gut microbiota, a new hope?

**DOI:** 10.1186/s12929-018-0476-7

**Published:** 2018-10-11

**Authors:** Matteo M Pusceddu, Melanie G Gareau

**Affiliations:** Department of Anatomy, Physiology and Cell Biology, School of Veterinary Medicine, University of California Davis, One Shield Avenue, Davis, CA USA

**Keywords:** Microbiota-gut-brain axis, Microbiome, Visceral pain, Probiotics, Prebiotics, FGID, IBS, Colitis

## Abstract

**Background:**

Visceral pain is a complex and heterogeneous disorder, which can range from the mild discomfort of indigestion to the agonizing pain of renal colic. Regulation of visceral pain involves the spinal cord as well as higher order brain structures. Recent findings have linked the microbiota to gastrointestinal disorders characterized by abdominal pain suggesting the ability of microbes to modulate visceral hypersensitivity and nociception to pain.

**Main body:**

In this review we describe the neuroanatomical basis of visceral pain signaling and the existing evidence of its manipulation exerted by the gut microbiota. We included an updated overview of the potential therapeutic effects of dietary intervention, specifically probiotics and prebiotics, in alleviating hypersensitivity to visceral pain stimuli.

**Conclusions:**

The gut microbiota dramatically impacts normal visceral pain sensation and affects the mechanisms mediating visceral nociception. Furthermore, manipulation of the gut microbiota using prebiotics and probiotics plays a potential role in the regulation of visceral pain disorders.

## Background

The increasing burden of visceral pain disorders has generated a growing interest by researchers and clinicians in studying the origins of pain from internal organs. Visceral pain is a complex and heterogeneous disorder which can range from the mild discomfort of indigestion to the agonizing pain of renal colic, typically disproportionately affecting more women than men [5, 10]. The most prevalent forms of visceral pain are categorized as functional gastrointestinal disorders (FGID) such as irritable bowel syndrome (IBS), which exceeds US$ 40 billion in medical costs and affects an estimated 10–15% of the US and European populations [62, 71]. Visceral pain disorders exert a tremendous pressure on the health care system and are associated with psychological distress, sleep disorders and sexual dysfunction, negatively impacting overall patient quality of life [35]. Moreover, both ageing and gender affect the progression of visceral pathology and pain, with IBS reported twice as frequently in women than in men [7].

**Fig. 1:**
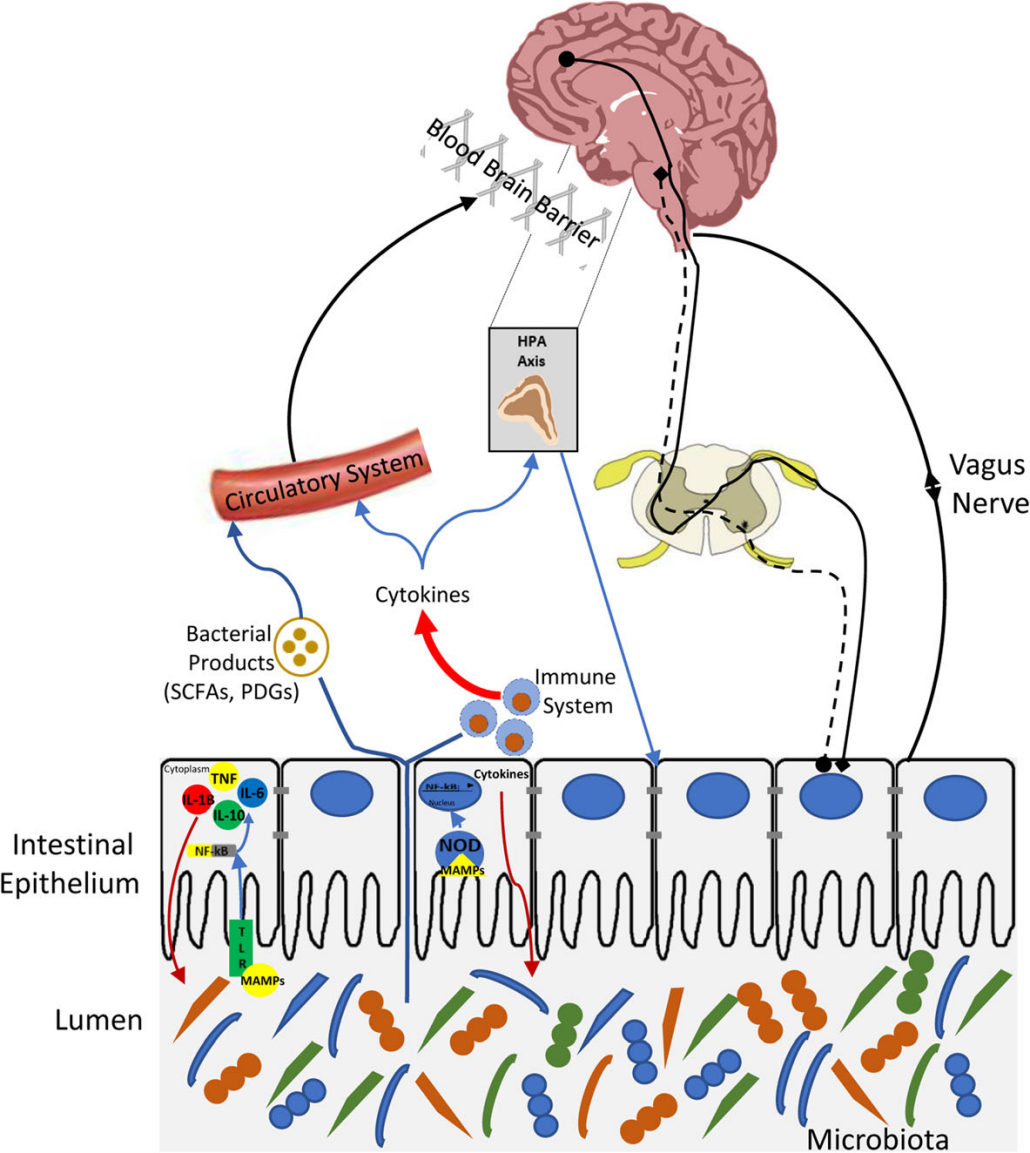
Gut microbiota-host interaction. Schematic representing the different ways of interaction between the microbiota and host. Painful stimuli sensed by nociceptors expressed at the nerve terminations project signals onto spinal nociceptive neurons located in the lateral neck of the dorsal horn of the spinal cord, which convey information to supraspinal centers. Here, the signal reaches several brain areas such as the thalamus, hypothalamus, limbic system, and cortex, which in concert code the afferent information and generate an efferent signal back to the periphery. The microbiota, which resides in the lumen of the gastrointestinal tract, can influence several factors involved in pain perception and its signaling such as the vagus nerve, cytokine production, corticosterone secretion, short chain fatty acids (SCFAs), and microbial metabolite release

The mechanisms involved in the perception of gastrointestinal pain and discomfort are complex. Stretch, inflammation, ischemia, pH, bacterial products, immune mediators, and neurotransmitters have all been associated with visceral pain [67]. Nociceptors, expressing transient receptor potential (TRP) at the nerve terminations, sense painful stimuli and project signals onto spinal nociceptive neurons located in the lateral neck of the dorsal horn of the spinal cord, which convey information to supraspinal centers (Fig. 1). Here, the signal reaches several brain areas such as thalamus, hypothalamus, limbic system and cortex, which in concert code the afferent information and generate an efferent signal back to the periphery [9]. Thus, the descending pathways modulate neuronal activity exerting either an inhibitory or a facilitatory effect on the sensation of pain. However, repeated or chronic nociceptors’ activation, due to chronic release of inflammatory mediators and pain signals following tissue injury, can lead to sensitization of the receptors and unpredictable bouts of visceral pain [32, 76]. For instance, substance P, serotonin, acetylcholine, prostaglandin 2, histamine, and cytokines are some of the mediators thought to play a role in the regulation of pain stimuli [76]. As alterations in the perception and maintenance of this type of pain involves multiple factors, making it challenging and often unsatisfactory in the choice and the development of adequate treatment options.

The microbiota has emerged as a novel target for the treatment of visceral pain. A correlation between visceral pain disorders, such as IBS, and microbial dysbiosis has been demonstrated in patients [19, 21]. Further evidence supports the role of bacterial, viral, and parasitic infections in triggering IBS symptoms. A recent systematic review and meta-analysis of 45 studies, comprising 21,421 individuals with enteritis, showed that the development of IBS was increased more than 10% up to at least 12 months post-infection. Moreover, the risk of IBS was found to be 4-fold higher than in individuals who did not have infectious enteritis, although heterogeneity among the studies were found. The increased risk of developing IBS was seen predominantly in women, as well as in individuals treated with antibiotics during the enteritis. [42]. Of interest, the improvement of visceral hypersensitivity through the use of certain beneficial probiotics and prebiotics has been recently proposed [26]. Moreover, significant enthusiasm has been generated following the potential benefits exerted by fecal material transplantation having been observed in patients with visceral pain [37, 59]. Therefore, the role of the intestinal microbiota has emerged as an essential player in the development of future therapeutic approaches for visceral pain.

## Gut microbiota development

The gut microbiome comprises more than 1000 species and 7000 strains dominated mainly by bacteria, but also includes viruses, protozoa, archaea and fungi [46]. This ecosystem occupies different niches in the human body, interacting with most, if not all, organs of the host throughout the lifespan. As first proposed by Tissier [73], colonization of the gut was assumed to commence at birth, making the human placenta an excellent sterile compartment for the growing offspring. However, the detection of a shared microbial signature between the placenta, amniotic fluid, and meconium suggests a direct maternal to infant transfer of microbiomes that starts in utero [43]. This maternal imprinting of the infant microbiota is then strengthened by breastfeeding during the first weeks of life giving shape to a much more complex microbiota in the offspring composed mostly by the genera Lactobacillus, Staphylococcus, Enterococcus, and Bifidobacterium [52]. The switch from breast milk to the introduction of solid food makes the microbiome gradually more complex, culminating in a more mature gut microbiota by 3 years of age [57]. Starting in the early stages of life, the microbiome establishes a long evolutionary symbiosis with the host, which influences essentially all organs, systems, as well as their functionality. For instance, the formation of a more mature microbiota early in life coincides with the development of the immune system, suggesting the microbiota is responsible for the priming of the immune system [4, 31].

From the gut, the microbiota can communicate with the central nervous system (CNS) forming a complex crosstalk between the gut, its microbiome, and the brain known as the microbiota-gut-brain (MGB) axis [17]. This bidirectional communication between the gut microbiota and the brain is believed to participate in the regulation of gastrointestinal homeostasis and affect CNS function including mood, cognition, and pain perception. The mechanisms by which the gut microbiota interacts with the host will be discussed thoroughly in this review article.

## Gut microbiota and its interaction with the host

The gastrointestinal (GI) tract is the most heavily colonized organ of the human body, which hosts an increasing microbial concentration from 10^1^ to 10^3^ cells up to 10^11^–10^12^ cells per gram of fecal contents in the stomach and in the colon, respectively [36]. Here the microbiota is recognized by the host by specific receptors expressed on different cells of the innate immune system, such as macrophages, neutrophils, NK cells, dendritic cells and intestinal epithelial cells. Specifically, microbe- or pathogen-associated molecular patterns (MAMPs or PAMPs), such as lipopolysaccharide (LPS) and peptidoglycans (PGN), are sensed by pattern recognition receptors (PRRs), including Toll-Like receptors (TLRs) and NOD-like receptors which are expressed on the host cell surface or in the cytosolic compartment of numerous cell types including immune cells [51]. The activation of PRRs triggers an enzymatic cascade leading to the synthesis and release of proinflammatory cytokines. In a chronically inflamed host, the integrity of the intestinal mucosal barrier is impaired facilitating bacterial infiltration across the gut and the migration of diverse bacterial antigens from the underlying lamina propria systemically via the blood. Therefore, following inflammation, a combination of cytokines and bacterial products, such as peptidoglycans and LPS, circulate into the blood, reaching several distant organs and systems including the CNS and the blood brain barrier (BBB). Whether cytokines can cross the BBB or not still needs to be clarified. However, evidence reveals that cytokines can influence brain areas and their functionality, suggesting a correlation exists between brain cytokines levels and psychiatric symptoms (including perception of pain), known as cytokine-induced sickness behavior [78]. Moreover, the heightened inflammatory tone induced by a leaky gut is also responsible for the activation of the hypothalamic-pituitary-adrenal (HPA) axis and consequently the release of corticosterone, the most potent stress hormone. This highlights the importance of the microbiota in influencing the neuroendocrine system [15]. Recent evidence indicates PGN can translocate into the brain and be sensed by PRRs within the CNS. [3]. Moreover, microglial control of astrocytes and CNS inflammation can be modulated by metabolites of dietary tryptophan produced by commensal bacteria, suggesting a novel signaling pathway that mediates the communication between the gut microbiota and the brain [65]. Other microbial products, specifically short chain fatty acids (SCFAs), can enter the blood and exert an effect centrally, influencing memory and cognition through epigenetic mechanisms [24, 45]. Furthermore, the microbiota is believed to influence function and metabolism of enteroendocrine cells, inducing the expression of several peptides, such as glucagon-like peptides (GLP) and peptide YY (PYY), which are known to control energy homeostasis, glucose metabolism, gut barrier function, and metabolic inflammation [8]. The microbiota is also capable of regulating the synthesis and release of several neurotransmitters in the GI tract. Microbial dependent-serotonin (5-HT) biosynthesis has emerged as a critical player, due to its implication in colonic function and GI disorders [34, 77]. For instance, lower mucosal 5-HT content, tryptophan hydroxylase (TPH) 1, and serotonin reuptake transporter (SERT) expression levels have been reported in some studies involving IBS patients [13, 25, 38]. Furthermore, exposure to selective serotonin reuptake inhibitor (SSRIs) in some cases have been shown to ameliorate IBS symptoms, such as enhanced orocecal transit and increased colonic motility [11, 72]. Moreover, the antagonism of specific 5-HT receptors abundantly expressed in the gut, such as 5-HT_3,_ has been shown to reduce visceral pain, slow colonic transit, and enhance small intestinal absorption [6]. Despite this evidence, the role of 5-HT signaling in the gut remains confusing and controversial, therefore further research is warranted [48]. In addition to 5-HT, the neurotransmitters γ-aminobutyric acid (GABA), dopamine (DA) and acetylcholine (ACh) are also synthesized in the lumen of the intestine by the microbiota and these neurotransmitters are believed to communicate with the brain via the vagus nerve [47]. It is also believed that the microbiota communicates with the CNS through the enteric nervous system (ENS) via vagal parasympathetic and sympathetic tracts [55]. A schematic representing the pathways of interaction between the microbiota and host is shown in Fig. 1.

## Visceral pain: Microbiota & Preclinical Studies

In recent years, preclinical studies have shed light on the role played by the microbiota in visceral pain. Studies using germ free mice (GF; mice raised without any exposure to microorganisms), have shown the commensal microbiota is necessary for development of an adequate pain sensitivity [2], which is blunted in response to several stimuli including bacterial LPS and interleukin (IL)-1β in GF mice [12]. Reestablishment of a normal microbiota through microbial transfer from conventional to GF mice has demonstrated that the microbiota is necessary for the restoration of normal excitability of gut sensory neurons [49]. Of note, fecal transplant from IBS patients reproduced certain features characteristics of IBS in GF mice, including hypersensitivity to colorectal distension, [14]. In another study, GF rats inoculated with the microbiota from patients with IBS developed abnormal gut fermentation mostly characterized by increased H2 excretion and sulfide production, [14] which have been reported in IBS [41, 69]. GF rodents represent a valuable tool for the investigation of visceral pain and related pathologies arising from intestinal dysbiosis.

### Probiotics in animal models

As an alternative to a GF state, chronic antibiotic administration is also used as a model to deplete the gut microbiota. Antibiotics can alter the innate mucosal immune system and attenuate visceral pain-related responses provoked by intracolonic capsaicin and intraperitoneal acetic acid administration in mice [1]. However, exposure to antibiotics during early life can also increase visceral sensitivity in adult rats, suggesting that alterations of the microbiota induced in specific time windows of life are crucial to the development of a sensitivity to pain [53].

Probiotics, bacteria that can confer beneficial effects onto the host following consumptionhave demonstrated improvements in animal models of visceral hypersensitivity. Despite these highly interesting findings, the mechanisms involved in mediating these benefits remain unkown [29] (Table 1). Live luminal administration of *Lactobacillus reuteri* (DSM 17938) and its conditioned medium dose-dependently reduced jejunal spinal nerve firing evoked by distension or capsaicin, with 80% of this response blocked by a specific transient receptor potential cation channel subfamily V member 1 (TRPV1) channel antagonist or in TRPV1 knockout mice [58]. *Lactobacillus acidophilus*-mediated analgesic effects function in the gut similarly to the effects of morphine, inducing upregulation of both opioid and cannabinoid receptors in rodents [66]. *Lactobacillus paracasei* administration blunted antibiotic-induced visceral sensitivity to colorectal distension (CRD) and increased substance P levels in the mice colon [74]. Interestingly, exposure to chronic stress has been used as a valuable rodent model of IBS and visceral sensitivity, suggesting the MGB axis serves as an important regulator of visceral pain. For instance, the neonatal maternal separation (MS) paradigm, which consists of separating murine pups from their mothers for 3 h per day for at least 10 days, induces several alterations related to visceral pain such as hypersensitivity to CRD, increased gut permeability, activation of the immune system, increased hypothalamic pituitary adrenal (HPA) axis activation and altered intestinal microbial composition [28, 54, 60, 70]. In this regard, a specific probiotics cocktail made of *L. helveticus* and *L. rhamnosus* reduced both macromolecular and paracellular permeability in MS [27]. The same probiotics treatment also ameliorated the MS-induced gut functional abnormalities and bacterial adhesion/penetration into the mucosa and blunted the HPA axis response [27]. *L. paracasei* and VSL#3, (composed of *B. longum*, *B. infantis*, *B. breve*, *L. acidophilus*, *L. casei*, *L. bulgaricus*, *L. plantarum*, and *Streptococcus salivarius*), were also able to reverse MS-induced hyperalgesia and allodynia during CRD and restored normal gut permeability [18, 23]. Moreover, VSL#3 was found to modulate the serotonergic system, specifically TPH1 expression levels, which is typically altered in IBS. VSL#3 was also shown to reduce gut permeability through upregulation of specific tight junction proteins (occluding, ZO-1) in a rat model of IBS induced by chronic intracolonic instillation of 4% acetic acid [16]. Similarly, both *L. helveticus* and *L. rhamnosus* administration were shown to restore the function of the intestinal barrier and increased the levels of tight junction proteins in two different animal models of colitis [44, 64].

**Table 1:** Effects of prebiotics and probiotics in preclinical studies

Animals	Treatment	Length of treatment	Outcomes	References
Adult male Swiss Webster	Live luminal *Lactobacillus reuteri* (DSM 17938)	9 days	DSM ↓ capsaicin-evoked (1) firing of spinal nerve action potentials and (2) Ca^2+^ increase in DRG neurons.	[58]
Sprague Dawley rats	*Lactobacillus acidophilus*	15 days	*L. acidophilus* ↑MOR1 and CB2 expression in intestinal epithelial cells restoring normal perception of visceral pain.	[66]
Female NIH Swiss mice and Balb/c mice	*Lactobacillus paracasei* (NCC2461)	10 days	*L. paracasei* ↓antibiotic-induced CRD hypersensitivity and SP immunolabelling in the myenteric plexus.	[74]
Sprague Dawley rats	*Lactobacillus rhamnosus and Lactobacillus helveticus*	15 days	Probiotics ↓ MS-induced CRD hypersensitivity, plasma CORT levels and short-circuit current in the gut.	[27]
Sprague Dawley rats	*Lactobacillus paracasei*	15 days	*L. paracasei* ↓ MS-induced CRD hypersensitivity.	[23]
Wistar rats	VSL#3	60 days	VSL#3 reversed MS-induced CRD hypersensitivity and alterations of i.e. TPH1, CCL2, NOS3, NTRK1, IL-10, TRPV4, gene expression levels.	[18]
C57BL/6 mice	*Lactobacillus rhamnosus and Lactobacillus helveticus*	15 days	Probiotics prevented c. rodentium-induced epithelial cell hyperplasia and reduction in cell proliferation as well as transcription of IL-10 and FOXP3.	[64]

*Abbreviations*: *MOR1* Opioid Receptor Mu, *CB2* Cannabinoid Receptor, *CRD* colorectal distension, *MS* maternal separation, *CORT* corticosterone, *TPH1* Tryptophan hydroxylase, *CCL2* C-C Motif Chemokine Ligand, *NOS3* nitric oxide synthase, *NTRK1* Neurotrophic Receptor Tyrosine Kinase, *IL-10* interleukin, *TRPV4* Transient Receptor Potential Cation Channel Subfamily V Member 4, *FOXP3* Forkhead Box P3

## Visceral pain: Microbiota & Clinical Studies

Intestinal dysbiosis has also been reported in individuals suffering from visceral pain, including IBS patients, making the microbiota itself a novel target for treatment [29, 61]. A reduction in the levels of Bifidobacterium, Lactobacillus [68] as well as alterations in the Firmicutes:Bacteroidetes ratio, which represent the most abundant phylum bacteria found within the human gut microbiome [63], have been identified in IBS patients. VSL#3 treatment has been shown to be effective in five small different randomized control trials (RCT) in IBS patients that fulfilled the Rome II or Rome III criteria. At least 6 weeks of VSL#3 treatment were necessary to observe improvements in symptomatology, such as reduced abdominal pain/discomfort, or improved abdominal bloating/gassiness, when compared to placebo [33, 39, 40, 50, 63]. A larger study involving 362 women with IBS demonstrated efficacy of *B. infantis* in reducing pain, bloating and improving bowel movements after 4 weeks of treatment compared to placebo [75]. Similarly, *L. rhamnosus* [30] and *L. plantarum* [20] both showed amelioration in abdominal pain and bloating together with reduced visceral pain in two different large RCT studies in IBS patients. *Escherichia coli* DSM 17252 has also showed improvements in 298 IBS patients compared to placebo. After 8 weeks of treatment, both abdominal pain and general pain scores were significantly ameliorated in the IBS group provided with probiotics [22]. One study showed beneficial effects of the prebiotic fructoligosaccharides (FOS) in patients affected by minor functional bowel disorders (FBD; Rome II criteria). After 6 weeks of treatment, 105 FBD patients showed reduced incidence and intensity of gastrointestinal symptoms over placebo [56]. Taken together, these studies highlight the potential for beneficial probiotics for the treatment of visceral pain.

The paucity of information coming from the accumulated clinical evidence to date limits our understanding on the efficacy of both prebiotics and probiotics in visceral pain (Table 2). Limitations are mostly due to inconsistencies within the studies, types of probiotics provided, length of the treatment and different types of pain disorders being treated. Nonetheless, the data to date suggests potential benefits exerted by specific probiotics and prebiotics in patients with visceral pain.

**Table 2:** Effects of prebiotics and probiotics in clinical studies

Participants	Treatment	Length of Trial	Outcomes	References
50 IBS children, Rome II criteria.	*Lactobacillus* GG vs placebo.	6 weeks	LGG ↓ incidence abdominal distention.	[50]
48 IBS patients, Rome II criteria.	VSL#3 vs placebo.	4 and 8 weeks	↓ flatulence and colonic transit.	[39]
30 Rome III FC patients; 30 controls.	VSL#3 vs placebo.	2 weeks	VSL#3 ↑complete spontaneous bowel movements.	[40]
59 IBS children.	VSL#3 vs placebo.	6 weeks	VSL#3 ↓ abdominal pain/discomfort, and bloating/gassiness.	[33]
104 children diagnosed with FAPD, IBS or FD.	*Lactobacillus* GG vs placebo.	4 weeks.	LGG treatment moderately improved abdominal pain.	[30]
105 FBD patients.	sc-FOS vs placebo.	6 weeks	sc-FOS ↓ intensity of digestive disorder symptoms ↑ quality of life, ↑ discomfort scores.	[56]

*Abbreviations*: *FBD* Functional Bowel Disorders, *FAPD* Functional Abdominal Pain Disorders, *FD* Functional Dyspepsia, *sc-FOS* short-chain Fructo-oligosaccharides

## Conclusions

Increasing evidence strongly indicates that the gut microbiota plays a pivotal role in the regulation of visceral pain. Its association with autonomic and emotional reactions and visceral function makes the gut microbiota an appealing target for novel pharmacological strategies against visceral pain in FGIDs, including IBS. Despite this, whether the microbiota is driving the abnormalities found in visceral pain and related pathologies remains to be resolved. Moreover, our knowledge on the crosstalk between the gut and brain and the mechanisms by which the microbiota could alleviate visceral pain is still in its early infancy. The provocative preclinical evidence on the influence of the microbiota in the regulation of visceral pain seems promising but still need to be confirmed clinically. Even though growing clinical research has found alleviation in the symptomatology of visceral pain after microbial manipulation with both prebiotics and probiotics, many lack power. Further studies with greater numbers of patients showing consistent results are warranted. Finally, whether fecal transplantation could be considered as a viable therapeutic option to modify the microbiota for benefit in visceral pain still needs to be confirmed.

## Abbreviations

5-HT: Serotonin
ACh: Acetylcholine
BBB: Blood brain barrier
CNS: Central nervous system
DA: Dopamine
ENS: Enteric nervous system
FBD: Functional bowel disorder
FGID: Functional gastrointestinal disease
GABA: Gamma-Aminobutyric acid
GF: Germ-free
GI: Gastrointestinal
GLP: Glucagon like peptide
HPA: Hypothalamic-pituitary-adrenal axis
IBS: Irritable bowel syndrome
IL: Interleukin
LPS: Lipopolysaccharide
MAMP: Microbial associated molecular pattern
MGB: Microbiota-gut-brain
MS: Maternal separation
PAMP: Pathogen associated molecular pattern
PGN: Peptidoglycan
PRR: Pattern recognition receptor
PYY: Peptide YY
RCT: Randomized control trial
SCFA: Short chain fatty acids
SERT: Serotonin reuptake transporter
SSRI: Serotonin selective reuptake inhibitor
TLR: Toll-like receptor
TPH: Tryptophan hydroxylase
TRP: Transient receptor potential
TRPV1: Transient receptor potential cation channel subfamily V member 1
ZO-1: Zonnula occuldens
